# A novel procalcitonin-based score for detecting sepsis among critically ill patients

**DOI:** 10.1371/journal.pone.0245748

**Published:** 2021-01-22

**Authors:** Tung-Lin Tsui, Ya-Ting Huang, Wei-Chih Kan, Mao-Sheng Huang, Min-Yu Lai, Kwo-Chang Ueng, Chih-Chung Shiao

**Affiliations:** 1 Institute of Medicine, Chung Shan Medical University, Taichung, Taiwan; 2 Intensive Care Unit, Department of Internal Medicine, Camillian Saint Mary’s Hospital Luodong, Luodong, Yilan, Taiwan; 3 Division of Cardiology, Department of Internal Medicine, Camillian Saint Mary’s Hospital Luodong, Luodong, Yilan, Taiwan; 4 Department of Nursing, Camillian Saint Mary’s Hospital Luodong, Luodong, Yilan, Taiwan; 5 Saint Mary’s Junior College of Medicine, Nursing and Management, Yilan, Taiwan; 6 Department of Nephrology, Department of Internal medicine, Chi-Mei Medical Center, Tainan, Taiwan; 7 Department of Biological Science and Technology, Chung Hwa University of Medical Technology, Tainan, Taiwan; 8 Department of laboratory medicine, Camillian Saint Mary’s Hospital Luodong, Luodong, Yilan, Taiwan; 9 School of Medicine, Chung Shan Medical University, Taichung, Taichung, Taiwan; 10 Division of Cardiology, Department of Internal Medicine, Chung Shan Medical University Hospital, Taichung, Taiwan; 11 Division of Nephrology, Department of Internal Medicine, Camillian Saint Mary’s Hospital Luodong, Luodong, Yilan, Taiwan; National Yang-Ming University, TAIWAN

## Abstract

**Background:**

Procalcitonin (PCT) has been widely investigated as an infection biomarker. The study aimed to prove that serum PCT, combining with other relevant variables, has an even better sepsis-detecting ability in critically ill patients.

**Methods:**

We conducted a retrospective cohort study in a regional teaching hospital enrolling eligible patients admitted to intensive care units (ICU) between July 1, 2016, and December 31, 2016, and followed them until March 31, 2017. The primary outcome measurement was the occurrence of sepsis. We used multivariate logistic regression analysis to determine the independent factors for sepsis and constructed a novel PCT-based score containing these factors. The area under the receiver operating characteristics curve (AUROC) was applied to evaluate sepsis-detecting abilities. Finally, we validated the score using a validation cohort.

**Results:**

A total of 258 critically ill patients (70.9±16.3 years; 55.4% man) were enrolled in the derivation cohort and further subgrouped into the sepsis group (n = 115) and the non-sepsis group (n = 143). By using the multivariate logistic regression analysis, we disclosed five independent factors for detecting sepsis, namely, “serum PCT level,” “albumin level” and “neutrophil-lymphocyte ratio” at ICU admission, along with “diabetes mellitus,” and “with vasopressor.” We subsequently constructed a PCT-based score containing the five weighted factors. The PCT-based score performed well in detecting sepsis with the cut-points of 8 points (AUROC 0.80; 95% confidence interval (CI) 0.74–0.85; sensitivity 0.70; specificity 0.76), which was better than PCT alone, C-reactive protein and infection probability score. The findings were confirmed using an independent validation cohort (n = 72, 69.2±16.7 years, 62.5% men) (cut-point: 8 points; AUROC, 0.79; 95% CI 0.69–0.90; sensitivity 0.64; specificity 0.87).

**Conclusions:**

We proposed a novel PCT-based score that performs better in detecting sepsis than serum PCT levels alone, C-reactive protein, and infection probability score.

## Background

Sepsis, defined as life-threatening organ dysfunction resulted from a dysregulated host response to infection, is a complex multifactorial syndrome carrying high health and economic burden worldwide [1–3]. In sepsis care, the accepted principle is an early diagnosis with prompt antibiotics therapy and infection source control before establishing organ dysfunction [4]. However, there is still lacking a gold standard for diagnosing sepsis, guiding therapy, and predicting prognoses for patients with sepsis [5].

Procalcitonin (PCT) is a 116-amino acid precursor polypeptide for calcitonin, which lowers serum calcium concentration. Besides the calcium homeostasis, the PCT also has some biological functions, including modulation of immunologic functions, influence on vasomotility and microcirculation, and alteration of cytokines expression during endotoxin shock [6, 7]. The PCT is released into the blood circulation and eliminated through the kidneys and liver [8].

In healthy individuals without an inflammatory situation, PCT is produced and converted to calcitonin within the C-cells in the thyroid gland, presenting very low serum PCT levels (< 0.1 ng/mL) [9]. When an inflammatory/infection occurs in the individuals, the PCT production process is alternatively and independently triggered by bacterial endotoxin and inflammatory cytokines and takes place in many extrathyroid organs, including liver, lung, kidney, pancreas, brain, heart, and small intestine [9, 10]. Under the inflammatory situation, PCT tends to rise rapidly within the first 3–4 hours after the event’s onset, peaks in the 6–12 hours, following a decrease after 24 hours and normalization within five days [11, 12].

PCT has been widely investigated as an infection biomarker with conflicting results [13–15]. Some studies demonstrated that PCT performs better to differentiate infectious from non-infectious illnesses than other sepsis biomarkers [16, 17]. The PCT value is associated with the severity of illness [18], and the change of PCT value links with prognoses in patients with severe infection [19, 20]. On the contrary, other investigations found that PCT cannot distinguish infectious from non-infectious systemic inflammatory response syndrome with high certainty [14] and does not correlate with mortality in patients with abdominal sepsis [21]. These conflicting results prohibit the universal consensus on the optimal use of PCT in the field of sepsis [22].

We hypothesized that serum PCT has a good detecting ability for sepsis in critically ill patients, and the sepsis-detecting ability is even better when PCT combines with other clinical variables. Thus, we conducted this study to build a novel PCT-based scoring system and evaluate the sepsis-detecting ability and prognosis-predictive ability of the proposed sepsis score.

## Materials and methods

### Study design and participants selection

This study was reviewed and approved by the Institutional Review Board of Saint Mary’s Hospital Luodong (approval # SMHIRB_105012). The study design conformed to the 1975 Declaration of Helsinki’s ethical guidelines, and the study was performed following the study protocol and relevant guidelines. The need for written informed consent was waived by the Institutional Review Board mentioned above because there was neither breach of privacy nor interference with clinical practice. The data were analyzed anonymously.

We conducted this retrospective cohort study in a regional teaching hospital, Saint Mary’s Hospital Luodong, in Taiwan. We enrolled eligible patients admitted to intensive care units (ICUs) between July 1, 2016, and December 31, 2016, and had serum PCT measured within 24 hours after ICU admission. The exclusion criteria included patients less than 18 years of age, along with those who had exposed surgeries or trauma within seven days before serum PCT measurement. For those with more than one hospitalization, only the first hospitalization was included in the current study. We followed these enrolled patients until March 31, 2017. The primary outcome measurement was the occurrence of sepsis.

Firstly, we randomized all the enrolled patients into the “derivation cohort” and the “validation cohort” with patient numbers of about 4:1. Then we categorized patients of the individual two cohorts into “sepsis group” and “non-sepsis group” according to the existence of sepsis. After obtaining these basic and clinical data, we tried to evaluate the sepsis-detecting abilities of PCT and other risk factors and subsequently combined these relevant factors to construct a PCT-based scoring system. Furthermore, we compared the sepsis-detecting abilities of the PCT-based score with other known biomarkers and scores. Finally, we applied the PCT-based score on the validation cohort to validate the score’s sepsis-detecting ability. In the current study, we took the existing sepsis biomarkers and scores and some relevant severity scores to compare the sepsis-detecting ability with the current PCT-based score because the clinical criteria for diagnosing sepsis (sepsis-3) [2] had taken the concept of organ dysfunction into sepsis diagnosis.

We made the diagnosis of sepsis according to the sepsis-3 diagnostic criteria, which include (1) suspected or documented infection, along with (2) evidence of organ dysfunction represented by an acute increase in the sequential organ failure assessment (SOFA) score of ≥ 2 points [2]. The delta SOFA (dSOFA) score was calculated by comparing SOFA scores obtained within 24 hours after ICU admission to the baseline SOFA scores. The baseline SOFA scores were defined as (1) the SOFA score obtained during the ward stay before ICU admission for those transferred from the ward, (2) the SOFA scores obtained in the most recent previous admission or outpatient clinics for those whose ICU admission was directly from the emergency department, or (3) zero point for those without previous medical data and were told to be well-being in the past.

### Covariates

The demographic data, comorbidities, clinical variables at ICU admission, length of stay in hospital and ICU, mechanical ventilators, noninvasive positive pressure ventilators (NIPPV), or vasopressors support in-hospital mortality were obtained from medical records.

The sepsis scores such as infection probability score (IPS) [23], as well as several severity scores including acute physiology and chronic health evaluation II (APACHE II), SOFA score [24] and quick SOFA (qSOFA) score, multiple organ dysfunction scores (MODS), and logistic organ dysfunction system (LODS) score, were also calculated.

### Quantitative measurement of biomarkers

All the laboratory examinations were performed in the central laboratory of the hospital. The serum PCT levels were measured using the sandwich principle of an automatic electrochemiluminescent immunoassay (COBAS E411, ROCHE, Switzerland) with the analytical measurement range of 0.02–100 ng/mL and detection limit of < 0.02 ng/mL. The serum C-reactive protein (CRP) levels were measured using the particle enhanced immunoturbidimetric assay (COBAS C501, ROCHE, Switzerland) with the analytical measurement range of 0.03–35.0 mg/dL and detection limit of 0.46 ng/mL.

### Statistical analysis

We performed statistical analyses using Scientific Package for Social Science (PASW Statistics for Windows, Version 22.0, Chicago: SPSS Inc.) and R 3.6.3 (R Foundation for Statistical Computing, Vienna, Austria, accessed https://www.r-project.org/) software. In all statistical analyses, a p ≤ 0.05 was considered statistically significant. Continuous variables experienced normality evaluation using the Kolmogorov-Smirnov test and the Shapiro-Wilk normality test [25]. The continuous variables with normal distribution were reported as mean ± standard deviation (SD) and compared using an independent t-test. The continuous variables with abnormal distribution were presented with a median [minimal, maximal] and compared using an independent t-test after log transformation [26] and confirmation as normal distribution by Q-Q plot. Categorical variables were expressed as case number (percentage) and compared using the chi-square test.

The multivariate logistic regression model with the conditional forward stepwise method was used to investigate the odds ratio (OR) and p-value for detecting sepsis. The elimination criterion for the multivariate analysis was set at p > 0.05. Using the G-Power with α set at 0.05 and OR set of 2.02, the logistic regression method has a calculated power of 1.00 in our study. The OR of 2.02 was estimated from simple logistic regression evaluating sepsis and PCT levels’ association in ordinal form.

We determined the independent risk factors for detecting sepsis and estimated the individual weights of these factors using the multivariate logistic regression model. The continuous variables (such as PCT level, albumin level, and neutrophil-lymphocyte ratio (NLR)) were transformed into categorical variables using the several cut-points proposed in the previous works [27, 28]. The individual variables’ scores were composed of the arithmetic sum of OR derived from the multivariate logistic regression analysis, including all independent risk factors after each numerical rounding.

Subsequently, we conducted a PCT-based scoring formula by the OR identified in the multivariate model. The Hosmer and Lemeshow Goodness-of-Fit test [29] was used for calibration of the model. Collinearity diagnosis [30] with tolerance value and the variance inflation factor were used to evaluate the correlation among sepsis’s independent risk factors.

Finally, we evaluated the sepsis-detecting abilities of the proposed PCT-based score and other biomarkers and scores by applying the receiver operating characteristic (ROC) curve analysis with an area under the ROC curve (AUROC). We compared the different AUROCs by the statistical method proposed by DeLong et al. [31]. Furthermore, we validated the results using the validation cohort.

## Results

During the enrollment period, we extracted 745 patients who had serum PCT measurement from the hospital database and excluded 415 patients who were aged less than 18 years, who had no ICU hospitalization, whose serum PCT was not measured within 24 hours after ICU admission, or who experienced surgery or trauma within seven days before serum PCT measurement. Finally, we enrolled 330 patients, including 258 patients (70.9±16.3 years, 55.4% men) in the derivation cohort, and 72 patients (69.2±16.7 years, 62.5% men) in the validation cohort. The basic characteristics and comorbid diseases of the two cohorts were comparable (S1 Table).

### Basic characteristics and clinical variables in the derivation cohort

The patients in the derivation cohort were categorized into a sepsis group (n = 115, 44.6%) and a non-sepsis group (n = 143, 55.4%). Compared to the non-sepsis group, the sepsis group had a significantly higher proportion of diabetes mellitus (DM) (47.0% versus 33.6%, p = 0.029) and infection (100.0% versus 13.3%). Besides, the sepsis group had a higher proportion of patients necessitating ventilator (37.4% versus 21.0%, p = 0.004) and vasopressor (45.2% versus 19.6%) (all p<0.001 unless otherwise denoted). Other variables, including basic characteristics, comorbid diseases, patient mix, reasons for ICU admission, and outcomes, were not statistically different between the two groups (Table 1).

**Table 1:** Basic characteristics and clinical variables in the derivation cohort.

	Total (n = 258)	Non-sepsis group (n = 143)	Sepsis group (n = 115)	p-value
**Basic characteristics**				
Age, years	70.9 ± 16.3	71.9 ± 16.1	69.7 ± 16.0	0.270
Gender, men	143 (55.4%)	75 (52.4%)	68 (59.1%)	0.283
Smoker	54 (20.9%)	23 (16.1%)	31 (27.0%)	0.078
Charlson’s score, points	3.8 ± 2.6	3.9 ± 2.7	3.8 ± 2.5	0.858
SOFA scores (baseline), points	2 [0, 9]	1 [0, 9]	2 [0, 8]	0.533
**Comorbid diseases**				
Hypertension	149 (57.8%)	80 (55.9%)	69 (60.0%)	0.512
Diabetes mellitus	102 (39.5%)	48 (33.6%)	54 (47.0%)	0.029
Coronal artery disease	58 (22.5%)	37 (25.9%)	21 (18.3%)	0.145
Heart failure	37 (14.3%)	24 (16.8%)	13 (11.3%)	0.212
Chronic lung disease	66 (25.6%)	38 (26.6%)	28 (24.3%)	0.684
Chronic kidney disease	76 (29.5%)	43 (30.1%)	33 (28.7%)	0.810
Cerebral vascular accident	83 (32.2%)	44 (30.8%)	39 (33.9%)	0.591
Liver cirrhosis	24 (9.3%)	11 (7.7%)	13 (11.3%)	0.321
Malignancy	39 (15.1%)	25 (17.5%)	14 (12.2%)	0.237
**Patient mix_medical patients**	245 (95.0%)	136 (95.1%)	109 (94.8%)	0.906
**Reasons for ICU admission**				0.086
Respiratory problems	74 (28.7%)	46 (32.2%)	28 (24.3%)	
Cardiovascular problems	37 (14.3%)	26 (18.2%)	11 (9.6%)	
Neurological problems	10 (3.9%)	7 (4.9%)	3 (2.6%)	
Gastroenterological problems	21 (8.1%)	12 (8.4%)	9 (7.8%)	
Nephrological problem	39 (15.1%)	17 (11.9%)	22 (19.1%)	
**With infection** ^#^	134 (51.9%)	19 (13.3%)	115 (100.0%)	<0.001
**Types of infection** *				
Pneumonia	42 (16.3%)	6 (4.2%)	36 (31.3%)	<0.001
Urinary tract infection	54 (20.9%)	9 (6.3%)	45 (39.1%)	<0.001
Blood stream infection	49 (19%)	4 (2.8%)	45 (39.1%)	<0.001
Skin infection	9 (3.5%)	1 (0.7%)	8 (7.0%)	0.006
Other infection	36 (14%)	4 (2.8%)	32 (27.8%)	<0.001
**Clinical variables and outcomes**				
With ventilator	73 (28.3%)	30 (21.0%)	43 (37.4%)	0.004
With NIPPV	67 (26.0%)	35 (24.5%)	32 (27.8%)	0.542
With vasopressor	80 (31.0%)	28 (19.6%)	52 (45.2%)	<0.001
Length of hospital stay, days	12 [1, 111]	12 [1, 88]	12 [1, 111]	0.765
Length of ICU stay, days	4 [1, 34]	4[1, 24]	4 [1, 34]	0.358
In-hospital mortality	73 (28.3%)	35 (24.5%)	38 (33.0%)	0.129

**Note:** Continuous variables with normal distribution were reported as mean ± standard deviation and compared using an independent t-test. Those with abnormal distribution were presented with a median [minimal, maximal] and compared using an independent t-test after log transformation and confirmation as normal distribution by Q-Q plot. Categorical variables were expressed as case number (percentage) and compared using the chi-square test.

^#^ suspected or documented infection

* We documented all types of infection if a patient had more than one type of infection.

**Abbreviations:** ICU = intensive care unit, NIPPV = noninvasive positive pressure ventilators, SOFA = sequential organ failure assessment.

At ICU admission, the sepsis group had a higher body temperature (36.7±1.2 versus 36.4±1.2°C, p = 0.038), heart rate (107.4±23.2 versus 100.1±26 beat/min, p = 0.019), and NLR (median, 11.4 versus 5.8). Meanwhile, the sepsis group had a lower mean arterial pressure (MAP) (82.3±21.7 versus 94.0±27.0 mmHg), albumin levels (2.9±0.6 versus 3.3±0.6 g/dL), the arterial partial pressure of carbon dioxide (PCO2) (33.7±15.2 versus 39.4±21.1 mmHg, p = 0.016), and arterial bicarbonate (HCO3) (18.7±7.1 versus 21.0±8.7 mEq/L, p = 0.024) compared to the non-sepsis group. Besides, the sepsis group also had significantly higher levels of most of the sepsis biomarkers, sepsis scores, and severity scores. These biomarkers and scores including PCT (median, 3.0 versus 0.4 ng/ml), CRP (median, 7.6 versus 2.5 mg/dL, p = 0.030), qSOFA score (1.6±0.7 versus 1.3±0.8 points, p = 0.003), SOFA score (8.3±3.7 versus 5.6±3.7 points), dSOFA score (median, 6 versus 3 points, p = 0.002), MODS (5.1±1.9 versus 4.2±1.7 points), LODS (5.0±3.0 versus 4.2±3.0 points, p = 0.032) and IPS (14.0±4.9 versus 11.0±5.9 points) (all p< 0.001 unless otherwise denoted) (Table 2). Of note, in the “non-sepsis groups (n = 143),” the 19 (13.3%) patients with infection did not have a SOFA increase ≥2 points, while those with an increased SOFA ≥2 points did not have “suspected or documented infection.”

**Table 2:** Clinical variables at intensive care unit admission in the derivation cohort.

	Total (n = 258)	Non-sepsis group (n = 143)	Sepsis group (n = 115)	p-value
**Vital signs**				
Glasgow Coma Scale, points	10.5 ± 4.4	10.7 ± 4.6	10.2 ± 4.3	0.410
Body temperature, °C	36.5 ± 1.2	36.4 ± 1.2	36.7 ± 1.2	0.038
Heart rate, beat/min	103.4 ± 25.0	100.1 ± 26.1	107.4 ± 23.2	0.019
Respiratory rate, breath/min	25.1 ± 9.4	24.4 ± 9.8	25.7 ± 8.8	0.262
Mean arterial pressure, mmHg	88.8 ± 25.4	94.0 ± 27.0	82.3 ± 21.7	<0.001
**Laboratory data**				
White blood cell, x10^3^/mL	13.5 ± 8.6	13.2 ± 9.4	13.8 ± 7.6	0.559
Neutrophils-lymphocyte ratio	8 [0.2, 96.0]	5.8 [0.4, 91.0]	11.4 [0.2, 96.0]	<0.001
Hemoglobin, g/dL	10.9 ± 2.8	10.9 ± 2.8	11.0 ± 2.7	0.876
Platelet, x10^3^/mL	217.8 ± 116.8	227.5 ± 120.4	205.7 ± 111.4	0.136
Blood urea nitrogen, mmol/L	32.7 [5.3, 210.7]	32.4 [5.3, 205.0]	34.5 [7.7, 210.7]	0.301
Creatinine, mmol/L	1.5 [0.3, 18.2]	1.3 [0.3, 18.2]	1.6 [0.3, 15.1]	0.552
eGFR, ml/min/1.73m^2^	44.2 [1.3, 557.8]	51.9 [1.3, 557.8]	41.7 [3.3, 238.3]	0.104
AST, units/L	31 [3.4, 1111.0]	30 [3.4, 1111.0]	35 [10.0, 947.0]	0.772
ALT, units/L	25 [1.0, 1891.0]	25 [1.0,1891.0]	28 [1.0,365.0]	0.432
Bilirubin (total), mg/dL	0.9 [0, 42.5]	0.9 [0.1, 42.5]	0.8 [0, 14.8]	0.283
Glucose, mg/dL	180.5 [11.0, 1139.0]	175.0 [18.0, 1139.0]	188.0 [11.0, 849.0]	0.849
Albumin, g/dL	3.1 ± 0.6	3.3 ± 0.6	2.9 ± 0.6	<0.001
Sodium, mmol/L	137.1 ± 9.6	138.0 ± 8.4	136 ± 10.8	0.083
Potassium, mEq/L	4.2 ± 1.1	4.3 ± 1.2	4.1 ± 1.0	0.304
Calcium, mEq/L	8.3 ± 1.0	8.4 ± 0.9	8.2 ± 1.1	0.257
PH	7.4 ± 0.1	7.3 ± 0.1	7.4 ± 0.1	0.243
PCO2, mmHg	36.8 ± 18.9	39.4 ± 21.1	33.7 ± 15.2	0.016
PO2, mmHg	101.3 ± 60.0	107.6 ± 65.3	93.6 ± 51.8	0.056
HCO3, mEq/L	20.0 ± 8.1	21.0 ± 8.7	18.7 ± 7.1	0.024
SatO2, %	89.3 ± 17.7	88.3 ± 19.9	90.5 ± 14.6	0.335
**Electrocardiography findings**				
Normal sinus rhythm	94 (36.4%)	59 (41.3%)	35 (30.4%)	0.073
Sinus tachycardia	104 (40.3%)	47 (32.9%)	57 (49.6%)	0.007
Atrial fibrillation	45 (17.4%)	27 (18.9%)	18 (15.7%)	0.497
Right bundle branch block	26 (10.1%)	16 (11.2%)	10 (8.7%)	0.508
**Sepsis biomarkers and severity scores**				
Procalcitonin, ng/mL	0.8 [0, 475.6]	0.4 [0, 70.2]	3.0 [0, 475.6]	<0.001
C-reactive protein, mg/dL	4.9 [0, 121.1]	2.5 [0, 121.1]	7.6 [0.1, 44.9]	0.030
APACHE-II, points	20.9 ± 8.2	20.8 ± 8.6	20.9 ± 7.8	0.902
qSOFA score, points	1.4 ± 0.8	1.3 ± 0.8	1.6 ± 0.7	0.003
SOFA score, points	6.8 ± 3.9	5.6 ± 3.7	8.3 ± 3.7	<0.001
dSOFA score, points	4 [0, 17]	3 [0, 17]	6 [2, 15]	0.002
MODS, points	4.6 ± 1.8	4.2 ± 1.7	5.1 ± 1.9	<0.001
LODS, points	4.6 ± 3.0	4.2 ± 3.0	5.0 ± 3.0	0.032
IPS, points	12.5 ± 5.6	11.0 ± 5.9	14.0 ± 4.9	<0.001

**Note:** Continuous variables with normal distribution were reported as mean ± standard deviation and compared using an independent t-test. Those with abnormal distribution were presented with a median [minimal, maximal] and compared using an independent t-test after log transformation and confirmation as normal distribution by Q-Q plot. Categorical variables were expressed as case number (percentage) and compared using the chi-square test.

**Abbreviations:** APACHE II = acute physiology and chronic health evaluation II, ALT = alanine aminotransferase, AST = aspartate aminotransferase, dSOFA = delta sequential organ failure assessment, eGFR = estimated glomerular filtration rate, HCO3 = bicarbonate, ICU = intensive care unit, IPS = infection probability score, LODS = logistic organ dysfunction score, MODS = multiple organ dysfunction scores, PCO2 = partial pressure of carbon dioxide, PO2 = partial pressure of oxygen, qSOFA = quick sequential organ failure assessment, SatO2 = oxygen saturation, SOFA = sequential organ failure assessment.

The ROC analysis demonstrated that the sepsis-detecting ability of serum PCT levels (AUROC 0.74, 95% confidence interval (CI) 0.68–0.80) were statistically better than all of the other biomarkers and scores related to sepsis and disease severity (S1 Fig).

### The PCT-based score and probability of sepsis

Next step, we put all the basic characteristics, comorbid disease, clinical variables (listed in Table 1), along with vital signs and laboratory data at ICU admission (listed in Table 2) for collinearity diagnosis. After selected these variables that passed the collinearity test into the multivariate logistic regression model, we disclosed five independent risk factors for detecting sepsis. These risk factors included higher serum PCT level (OR = 1.0, 95%CI = 1.0–1.0, p = 0.002), higher NLR (OR = 1.0, 95%CI = 1.0–1.0), lower serum albumin level (OR = 0.3, 95%CI = 0.2–0.5), with DM (OR = 2.4, 95%CI = 1.3–4.4), and with vasopressor (OR = 2.1, 95%CI = 1.1–4.0, p = 0.003) (all p<0.001 except otherwise denoted) (Table 3).

**Table 3:** The independent factors for detecting sepsis.

Variables	Odds Ratio	95% Confidence Interval	p-value
**Procalcitonin level** ^**1**^	1.0	1.0–1.1	0.002
**Albumin level** ^**1**^	0.3	0.2–0.5	<0.001
**Neutrophil-lymphocyte ratio** ^**1**^	1.0	1.0–1.1	<0.001
**Diabetes mellitus**			
without	Reference		
with	2.4	1.3–4.4	<0.001
**Vasopressor**			
without	Reference		
with	2.1	1.1–4.0	0.003

**Note:** These independent risk factors were determined using the multivariate logistic regression model. The elimination criterion of the predictors was set at p > 0.05. Bootstrap approached for logistic regression with 2000 resampling. The Hosmer and Lemeshow test demonstrated an adequate calibration of the proposed model (goodness-of-fit statistic 4.10 with 8 degrees of freedom, p-value = 0.848).

^1^denots an increment of one unit.

**Fig 1:**
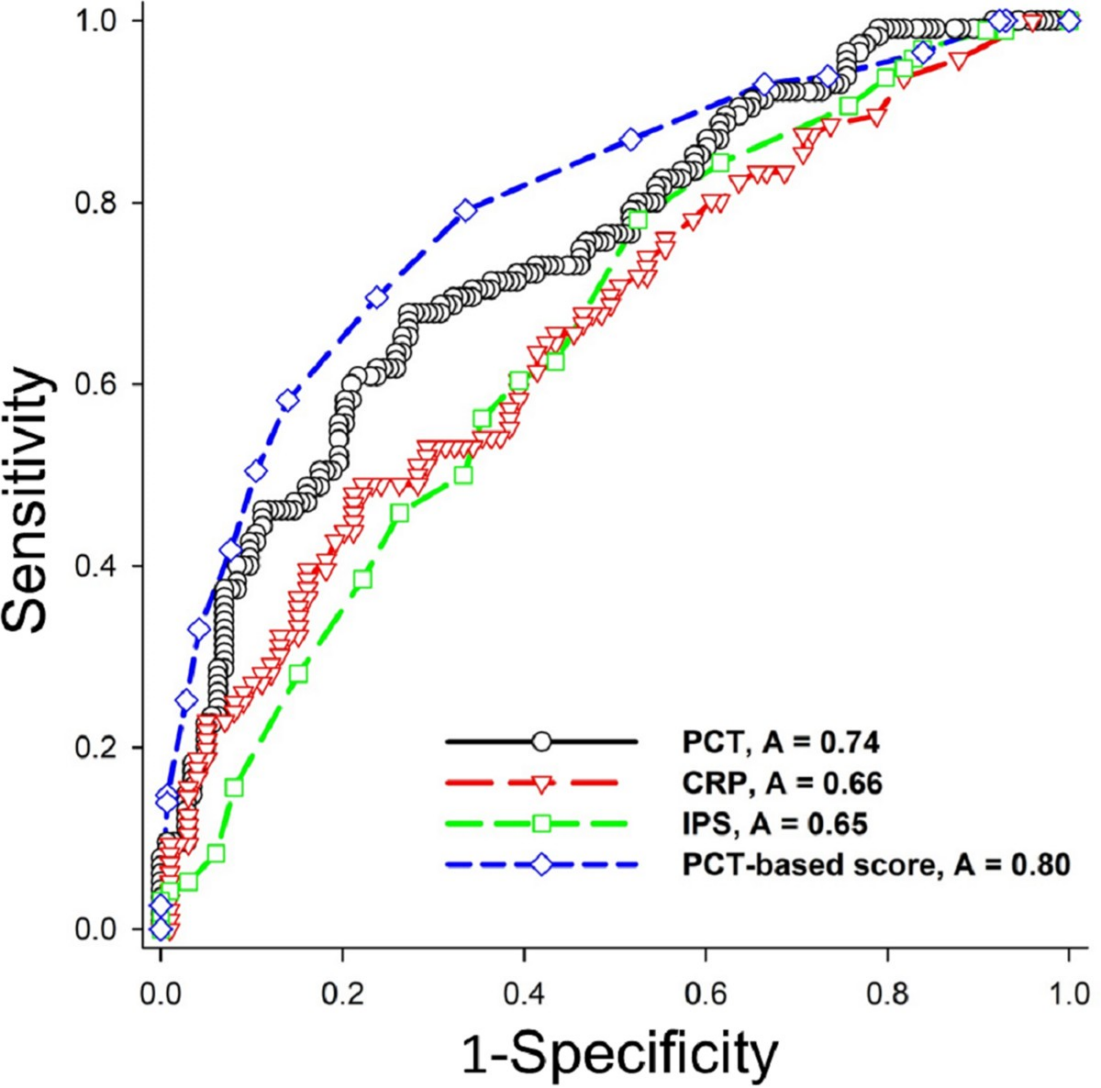
Comparison of sepsis-detecting abilities among the PCT-based score and other known sepsis biomarkers and scores. Procalcitonin (PCT)-based score (dashed blue line) had better predictability [area under the receiver operating characteristic curve (AUROC) = 0.80, 95% confidence interval = 0.74–0.85, Sensitivity = 0.70, Specificity = 0.76, Positive Predictive Value = 0.70, Negative Predictive Value = 0.76] than PCT (solid black line) (AUROC = 0.74), C-reactive protein (CRP) (dashed red line) (AUROC = 0.66) and infection probability score (IPS) (dashed green line) (AUROC = 0.65).

Subsequently, we combined the five weighted risk factors to constructed a PCT-based scoring system with a total score ranged from 0 to 17 points (Table 4). The PCT-based score of all patients in the derivation cohort was 7.4±3.9 points, which was significantly higher in the sepsis group (9.6±3.7 points) than the non-sepsis group (5.6± 3.1 points) (p<0.001). Finally, the ROC curve analysis demonstrated that, when setting a cut-point of 8 points, the PCT-based score had a good sepsis-detecting ability of the (AUROC 0.80, 95%CI 0.74–0.85, sensitivity 0.70, specificity 0.76), which were significantly better than the PCT level alone (p = 0.020), CRP (p<0.001), and IPS (p<0.001) (Fig 1, Table 5(A) and S2 Table) The Hosmer and Lemeshow test confirmed the adequate calibration of the proposed model (goodness-of-fit statistic 4.10 with 8 degrees of freedom, p = 0.848).

**Table 4:** The formula of the PCT-based score for detecting sepsis.

Variables	Point
**Procalcitonin level**	
< 0.5 ng/mL	+ 0
0.5–2.0 ng/mL	+ 1
2.0–10.0 ng/mL	+ 3
> 10.0 ng/mL	+ 5
**Albumin level**	
≧3.5 mg/dl	+ 0
2.5–3.4 mg/dl	+ 3
<2.5 mg/dl	+ 5
**Neutrophils-lymphocyte ratio**	
< 5	+ 0
5–10	+ 2
>10	+ 3
**Diabetes mellitus**	
without	+ 0
with	+ 2
**Vasopressor**	
without	+ 0
with	+ 2
**Total scores (0–17 points)**	

**Note:** The formula was conducted using the odds ratio identified in the multivariate model. The individual factors’ scores were composed of the arithmetic sum of the odds ratio derived from logistic regression analysis, including all independent risk factors after each numerical rounding.

**Table 5:** Comparisons of the sepsis-detecting abilities of the PCT-based score between the derivation cohort (A) and validation cohort (B).

	Cut-points	Area under curve (95%CI)	Sensitivity (95%CI)	Specificity (95%CI)	Positive predictive value (95%CI)	Negative predictive value (95%CI)
**(A) In derivation cohort**	8	0.80	0.70	0.76	0.70	0.76
		(0.74–0.85)	(0.61–0.78)	(0.69–0.83)	(0.62–0.79)	(0.69–0.83)
**(B) In validation cohort**	8	0.79	0.64	0.87	0.81	0.74
		(0.69–0.90)	(0.47–0.80)	(0.77–0.98)	(0.66–0.96)	(0.61–0.87)

**Abbreviations:** CI = confidence interval.

### Validation of the results by the validation cohort

Finally, we did the validation work using the validation cohort, including 33 patients (45.8%) in the sepsis group and 39 (54.2%) in the non-sepsis group. The sepsis group in the validation cohort had significantly higher proportion of DM (54.5% versus 28.2%, p = 0.023) and infection (100.0% versus 15.4%, p<0.001). Other basic and clinical variables were not statistically different between the two groups (S3 Table).

Using the ROC analysis, we validated the good sepsis-detecting ability of the PCT-based score in the validation cohort (cut-point: 8 points; AUROC 0.79, 95%CI 0.69–0.90, sensitivity 0.64, specificity 0.87) (Table 5). The results supported the novel PCT-based score as a good tool for detecting sepsis among critically ill patients.

## Discussion

To the best of our knowledge, this is the first study proposing a novel PCT-based score with an optimal sepsis-detecting ability, which was better than PCT, CRP, and IPS. To avoid the bias of varied practice patterns from different medical staff groups, we conducted an independent validation cohort by randomizing patients from the same total enrolled patients as the derivation cohort instead of enrolling another group of patients at another period.

### Components in the current PCT-based score

This PCT-based score was composed of five predictors, including higher serum PCT level, higher NLR, lower albumin level, DM, and necessitating vasopressor.

The PCT is a widely investigated biomarker in sepsis because of the short time between stimulus and PCT induction and its long half-life [11, 12]. PCT has high sensitivity and specificity to distinguish sepsis from a systemic inflammatory response syndrome of non-infectious origin, and an elevated PCT level is disclosed to associate with sepsis in hospitalized patients independently [32]. Furthermore, an increasing body of evidence shows that PCT is a useful tool for not only diagnosing sepsis [14, 15] but also guiding antibiotic treatment [33] and predicting prognosis [13–15]. These results are in line with the findings of the current study.

It is well known that individuals with DM have a higher risk of various acute and chronic infections than those without DM [34]. In diabetic patients, the persistent hyperglycemia results in abnormal metabolic changes, and the subsequently increased superoxide production and activation of inflammatory pathways [35]. These changes cause impairment of both the fast-acting innate immune defenses and the adaptive immune system [34]. It is reasonable to add comorbidity as a component of a scoring system. On famous and well-recognized example is the APACHE II score, which contains some comorbidity as components. Practically, adding DM into the current proposed score improves the ability to detect sepsis of the score. NLR is also a biomarker correlated with systemic inflammation and poor prognosis in the settings of acute bacterial infection [36], viral infection [37], or even tumors [38]. Interestingly, a recent study reported that Covid-19 patients with DM had a higher PCT level and higher NLR than those without DM [39]. The finding is suggestive of an association between infection and the above three factors.

Low serum albumin was an independent risk factor for sepsis in the current study. We provided two possible explanations for this finding. (1) Hypoalbuminemia is a clinical indicator of malnutrition, a well-known risk for increased infection risk, and unpleased patients’ prognoses across various clinical settings [40]. (2) Hypoalbuminemia results from and reflects the inflammatory and infection state. The pathophysiology behind hypoalbuminemia in these situations includes decreased protein synthesis, decreased half-life, and the total amount of serum albumin, increased capillary permeability, the volume of distribution, and expression vascular endothelial growth factor [41]. Lastly, the need for vasopressor support reflects an inadequate hemodynamic state, which is also a component in SOFA score and IPS playing to indicate cardiovascular system failure [23]. The current study underscored the role of needing vasopressor support for predicting sepsis.

### Comparisons of the sepsis scores

IPS [23], a score containing all SOFA score variables along with some vital signs and laboratory variables, has excellent value for diagnosing infection and dynamic evaluating antibiotic therapy response [16]. The concept of IPS consists of the primary concern of sepsis-3 criteria that takes “organ dysfunction” as an essential requirement for sepsis diagnosis. It is worth mentioning that only one component (i.e., vasopressor) in the current PCT-based score overlaps with SOFA score and IPS components. Other components of the PCT-based score include some laboratory variables instead of vital signs as in the IPS (S4 Table). In the current study, we found that the proposed PCT-based score performed better in detecting sepsis than IPS. This result contributes to the critical care community and provides a valuable reference for choosing variables for the generation of sepsis definition in the future.

## Limitations

Some potential limitations should be addressed. Firstly, the single-centered retrospective study design of the current study was subject to bias. Although we have successfully validated the sepsis-detecting ability of the proposed PCT-based score using an independent validation cohort, the internal validation strategies could not assure the generalization of the findings to other patient populations whose basic characteristics were different from the current derivation and validation cohorts (ex: those with younger age or fewer comorbidities). Secondly, the study’s included patients were selected by the criteria “existence of serum PCT measurements within 24 hours after ICU admission.” Thirdly, the patient population in the current study was mainly critically ill medical patients. The results might not be suitable to apply to other populations, such as surgical or trauma patients or those who are not critically ill. Fourthly, we designed to evaluate the association between serum PCT measurement at initial ICU admission and sepsis. The serial changes of serum PCT levels after management were not taken into consideration.

Further multicentered researches are encouraged to investigate the factors affecting serum PCT levels in the septic patient and to explore the relationship between the serial levels of serum PCT and patients’ outcomes.

## Conclusions

The current study proposed a novel PCT-based score, which performs better in detecting sepsis than the existing sepsis biomarkers and scores.

## Supporting information

S1 Fig Comparison of sepsis-detecting ability among biomarkers and severity scores. Procalcitonin (PCT) (solid black line) had the most significant area under the curve. Other biomarkers or severity scores with the gradual decreasing area under curve included delta sequential organ failure assessment (dSOFA) score (dashed green line), serum CRP (dashed red line), infection probability score (IPS) (dashed-double dot orange line), multiple organ dysfunction scores (MODS) (dashed grass green line), quick sequential organ failure assessment (qSOFA) (dashed blue line), logistic organ dysfunction score (LODS) (dashed-dot pink line), and acute physiology and chronic health evaluation II (APACHE II) (solid purple line).
(DOCX)

S1 Table Characteristics of the patients in the derivation and validation cohorts. **Note:** Continuous variables with normal distribution were reported as mean ± standard deviation and compared using an independent t-test. Those with abnormal distribution were presented with a median [minimal, maximal] and compared using an independent t-test after log transformation and confirmation as normal distribution by Q-Q plot. Categorical variables were expressed as case number (percentage) and compared using the chi-square test. ^#^ suspected or documented infection. * We documented all types of infection if a patient had more than one type of infection. **Abbreviations:** ICU = intensive care unit, SOFA = sequential organ failure assessment.
(DOCX)

S2 Table Comparisons of the procalcitonin-based score with other biomarkers and scores. **Abbreviations:** APACHE II = acute physiology and chronic health evaluation II, CRP = C-reactive protein, dSOFA = delta sequential organ failure assessment, IPS = infection probability score, LODS = logistic organ dysfunction score, MODS = multiple organ dysfunction scores, PCT = procalcitonin, qSOFA = quick sequential organ failure assessment.
(DOCX)

S3 Table Basic characteristics and clinical variables in the validation cohort. **Note:** Continuous variables with normal distribution were reported as mean ± standard deviation and compared using an independent t-test. Those with abnormal distribution were presented with median (range) and compared using an independent t-test after log transformation and confirmation as normal distribution by Q-Q plot. Categorical variables were expressed as case number (percentage) and compared using the chi-square test. ^#^ suspected or documented infection. * we counted all types of infection if a patient had more than one type of infection. **Abbreviations:** SOFA = sequential organ failure assessment.
(DOCX)

S4 Table Comparisons of the components in the scores. **Abbreviations:** IPS = infection probability score; PaO2/FiO2 = partial pressure of oxygen divided by the fraction of inspired oxygen; PCT = procalcitonin; SOFA = sequential organ failure assessment.
(DOCX)

S1 Dataset (XLSX)

## Abbreviations

ALT: alanine aminotransferase
ANOVA: analysis of variance
APACHE II: acute physiology and chronic health evaluation II
AST: aspartate aminotransferase
AUROC: area under the receiver operating characteristic curve
B: coefficient
CI: confidence interval
CLD: chronic lung disease
CRP: C-reactive protein
DM: diabetes mellitus
dSOFA: delta sequential organ failure assessment
eGFR: estimated glomerular filtration rate
HCO3: bicarbonate
ICU: intensive care unit
IPS: infection probability score
LODS: logistic organ dysfunction system
MAP: mean arterial pressure
MODS: multiple organ dysfunction score
NLR: neutrophil-lymphocyte ratio
NIPPV: noninvasive positive pressure ventilators
OR: odds ratio
PO2: partial pressure of oxygen
PaO2/FiO2: partial pressure of oxygen divided by the fraction of inspired oxygen
PCO2: partial pressure of carbon dioxide
PCT: procalcitonin
qSOFA: quick sequential organ failure assessment
ROC: receiver operating characteristic
SatO2: oxygen saturation
SD: standard deviation
SOFA: sequential organ failure assessment
