# Supplementary material for: A novel procalcitonin-based score for detecting sepsis among critically ill patients
Source: PLoS One. 2021 Jan 22;16(1):e0245748. doi: 10.1371/journal.pone.0245748 (PMC7822524; doi:10.1371/journal.pone.0245748)

**S1 Fig. Comparison of sepsis-detecting ability among biomarkers and severity scores**

**Note:** Procalcitonin (PCT) (solid black line) had the most significant area under the curve.

Other biomarkers or severity scores with the gradual decreasing area under curve included

delta sequential organ failure assessment (dSOFA) score (dashed green line), serum CRP (dashed red line), infection probability score (IPS) (dashed-double dot orange line), multiple organ dysfunction scores (MODS) (dashed grass green line), quick sequential organ failure assessment (qSOFA) (dashed blue line), logistic organ dysfunction score (LODS) (dashed-dot pink line), and acute physiology and chronic health evaluation II (APACHE II) (solid purple line).


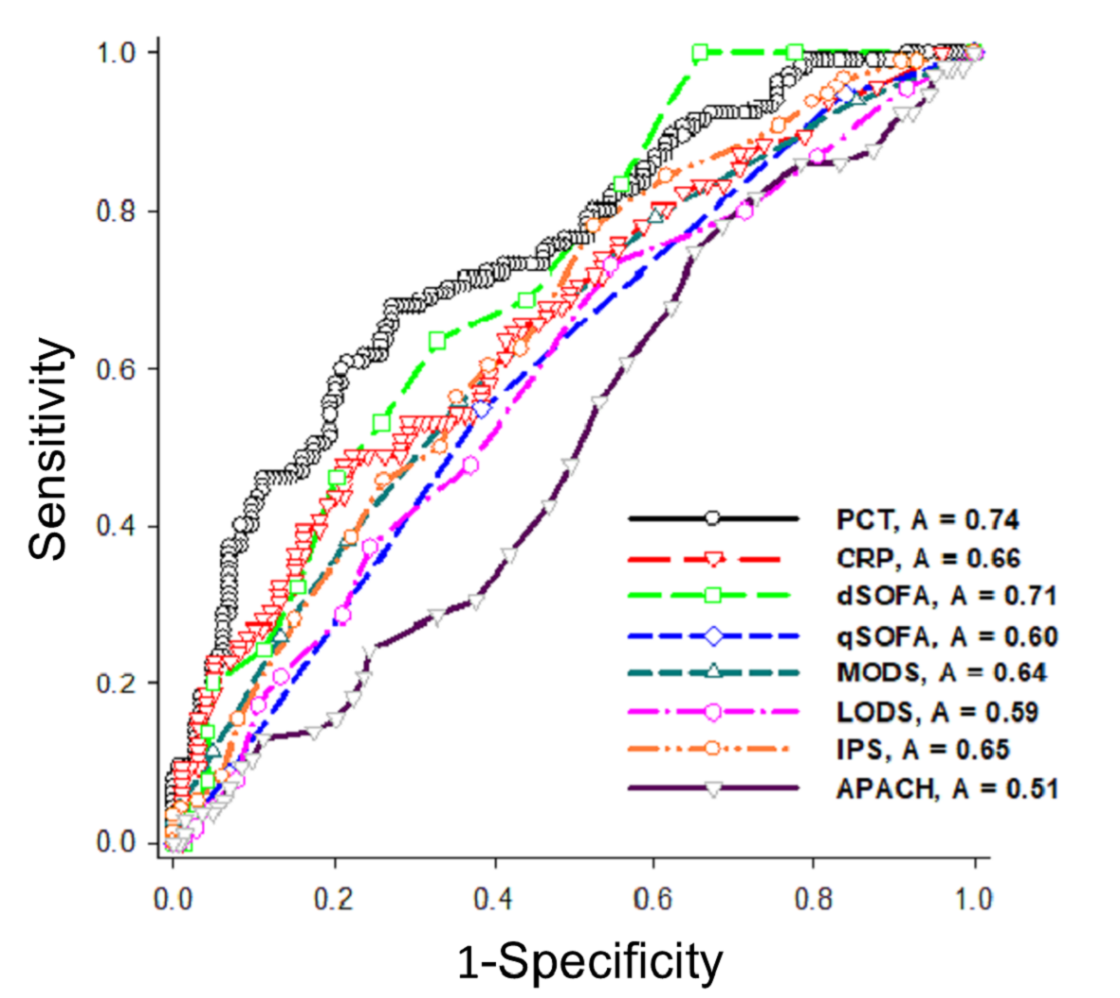

Supplement: S1 Fig — Procalcitonin (PCT) (solid black line) had the most significant area under the curve. Other biomarkers or severity scores with the gradual decreasing area under curve included delta sequential organ failure assessment (dSOFA) score (dashed green line), serum CRP (dashed red line), infection probability score (IPS) (dashed-double dot orange line), multiple organ dysfunction scores (MODS) (dashed grass green line), quick sequential organ failure assessment (qSOFA) (dashed blue line), logistic organ dysfunction score (LODS) (dashed-dot pink line), and acute physiology and chronic health evaluation II (APACHE II) (solid purple line). (DOCX) [file pone.0245748.s001.docx]
