# Supplementary material for: A novel procalcitonin-based score for detecting sepsis among critically ill patients
Source: PLoS One. 2021 Jan 22;16(1):e0245748. doi: 10.1371/journal.pone.0245748 (PMC7822524; doi:10.1371/journal.pone.0245748)
Supplement: S1 Table — Note: Continuous variables with normal distribution were reported as mean ± standard deviation and compared using an independent t-test. Those with abnormal distribution were presented with a median [minimal, maximal] and compared using an independent t-test after log transformation and confirmation as normal distribution by Q-Q plot. Categorical variables were expressed as case number (percentage) and compared using the chi-square test. # suspected or documented infection. * We documented all types of infection if a patient had more than one type of infection. Abbreviations: ICU = intensive care unit, SOFA = sequential organ failure assessment. (DOCX) [file pone.0245748.s002.docx]

**S1 Table. Characteristics of the patients in the derivation and validation cohorts**

|  | **Derivation cohort**  **(n=258)** | **Validation cohort**  **(n=72)** |
| --- | --- | --- |
| **Basic characteristics** |  |  |
| Age, years | 70.9 ± 16.3 | 69.2 ± 16.7 |
| Gender, men | 143 (55.4 %) | 45 (62.5 %) |
| Smoker | 54 (20.9 %) | 18 (25.0 %) |
| Charlson’s score | 3.8 ± 2.6 | 3.4 ± 2.7 |
| SOFA scores (baseline), points | 2 [0, 9] | 1 [0, 6] |
| **Comorbid disease** |  |  |
| Hypertension | 149 (57.8 %) | 40 (55.6 %) |
| Diabetes mellitus | 102 (39.5 %) | 29 (40.3 %) |
| Coronal artery disease | 58 (22.5 %) | 15 (20.8 %) |
| Heart failure | 37 (14.3 %) | 10 (13.9 %) |
| Chronic lung disease | 66 (25.6 %) | 16 (22.2 %) |
| Chronic kidney disease | 76 (29.5 %) | 22 (30.6 %) |
| Cerebral vascular accident | 83 (32.2 %) | 21 (29.2 %) |
| Liver cirrhosis | 24 (9.3 %) | 4 (5.6 %) |
| Malignancy | 39 (15.1 %) | 5 (6.9 %) |
| **Patient mix_medical patients** | 245 (95.0 %) | 65 (90.3%) |
| **Reasons for ICU admission** |  |  |
| Respiratory problems | 74 (28.7 %) | 24 (33.3 %) |
| Cardiovascular problems | 37 (14.3 %) | 8 (11.1 %) |
| Neurological problems | 10 (3.9 %) | 4 (5.6 %) |
| Gastroenterological problems | 21 (8.1 %) | 5 (6.9 %) |
| Nephrological problem | 39 (15.1 %) | 12 (16.7 %) |
| **With infection ^＃^** | 134 (51.9 %) | 39 (54.2 %) |
| **Types of infection *** |  |  |
| Pneumonia | 42 (16.3 %) | 10 (13.9 %) |
| Urinary tract infection | 54 (20.9 %) | 11 (15.3 %) |
| Blood stream infection | 49 (19 %) | 23 (31.9 %) |
| Skin infection | 9 (3.5 %) | 4 (5.6 %) |
| Other infection | 36 (14 %) | 9 (12.5 %) |

**Note:** Continuous variables with normal distribution were reported as mean ± standard deviation and compared using an independent t-test. Those with abnormal distribution were presented with a median [minimal, maximal] and compared using an independent t-test after log transformation and confirmation as normal distribution by Q-Q plot. Categorical variables were expressed as case number (percentage) and compared using the chi-square test.

^＃^ suspected or documented infection

***** We documented all types of infection if a patient had more than one type of infection.

**Abbreviations:** ICU= intensive care unit, SOFA= sequential organ failure assessment,
