# Supplementary material for: A novel procalcitonin-based score for detecting sepsis among critically ill patients
Source: PLoS One. 2021 Jan 22;16(1):e0245748. doi: 10.1371/journal.pone.0245748 (PMC7822524; doi:10.1371/journal.pone.0245748)
Supplement: S2 Table — Abbreviations: APACHE II = acute physiology and chronic health evaluation II, CRP = C-reactive protein, dSOFA = delta sequential organ failure assessment, IPS = infection probability score, LODS = logistic organ dysfunction score, MODS = multiple organ dysfunction scores, PCT = procalcitonin, qSOFA = quick sequential organ failure assessment. (DOCX) [file pone.0245748.s003.docx]

**S2 Table. Comparisons of the procalcitonin-based score with other biomarkers and scores**

|  | **Area Difference** | **Standard Error** | **95% Confidence Interval** | **Chi-Square, DF = 1** | **p-value** |
| --- | --- | --- | --- | --- | --- |
| PCT-based scores versus PCT | 0.054 | 0.023 | 0.01 – 0.10 | 5.4 | 0.020 |
| PCT-based scores versus CRP | 0.136 | 0.036 | 0.07 – 0.21 | 14.8 | < 0.001 |
| PCT-based scores versus IPS | 0.145 | 0.037 | 0.07 – 0.22 | 15.3 | < 0.001 |

**Note:** We compared the different areas under the receiver operating characteristic curves by the statistical method proposed by DeLong et al. [31].

**Abbreviations:** APACHE II= acute physiology and chronic health evaluation II, CRP= C-reactive protein, dSOFA= delta sequential organ failure assessment, IPS= infection probability score, LODS= logistic organ dysfunction score, MODS= multiple organ dysfunction scores, PCT= procalcitonin, qSOFA= quick sequential organ failure assessment.
