# Supplementary material for: A novel procalcitonin-based score for detecting sepsis among critically ill patients
Source: PLoS One. 2021 Jan 22;16(1):e0245748. doi: 10.1371/journal.pone.0245748 (PMC7822524; doi:10.1371/journal.pone.0245748)
Supplement: S3 Table — Note: Continuous variables with normal distribution were reported as mean ± standard deviation and compared using an independent t-test. Those with abnormal distribution were presented with median (range) and compared using an independent t-test after log transformation and confirmation as normal distribution by Q-Q plot. Categorical variables were expressed as case number (percentage) and compared using the chi-square test. # suspected or documented infection. * we counted all types of infection if a patient had more than one type of infection. Abbreviations: SOFA = sequential organ failure assessment. (DOCX) [file pone.0245748.s004.docx]

**S3 Table. Basic characteristics and clinical variables in the validation cohort**

|  | **Total**  **(n=72)** | **Non-sepsis group**  **(n=39)** | **Sepsis group**  **(n=33)** | **p-value** |
| --- | --- | --- | --- | --- |
| **Basic characteristics** |  |  |  |  |
| Age, years | 69.2 ± 16.7 | 69.2 ± 16.9 | 69.2 ± 16.7 | 0.998 |
| Gender, men | 45 (62.5 %) | 26 (66.7 %) | 19 (57.6 %) | 0.427 |
| Smoker | 18 (25.0 %) | 12 (30.8 %) | 6 (18.2 %) | 0.173 |
| Charlson’s score | 3.4 ± 2.7 | 2.9 ± 2.6 | 4.0 ± 2.7 | 0.089 |
| SOFA scores (baseline), points | 1 [0, 6] | 1 [0, 5] | 1 [0, 6] | 0.476 |
| **Comorbid disease** |  |  |  |  |
| Hypertension | 40 (55.6 %) | 22 (56.4 %) | 18 (54.5 %) | 0.874 |
| Diabetes mellitus | 29 (40.3 %) | 11 (28.2 %) | 18 (54.5 %) | 0.023 |
| Coronal artery disease | 15 (20.8 %) | 8 (20.5 %) | 7 (21.2 %) | 0.942 |
| Heart failure | 10 (13.9 %) | 7 (17.9 %) | 3 (9.1 %) | 0.279 |
| Chronic lung disease | 16 (22.2 %) | 6 (15.4 %) | 10 (30.3 %) | 0.129 |
| Chronic kidney disease | 22 (30.6 %) | 10 (25.6 %) | 12 (36.4 %) | 0.325 |
| Cerebral vascular accident | 21 (29.2 %) | 9 (23.1 %) | 12 (36.4 %) | 0.217 |
| Liver cirrhosis | 4 (5.6 %) | 3 (7.7 %) | 1 (3 %) | 0.390 |
| Malignancy | 5 (6.9 %) | 4 (10.3 %) | 1 (3 %) | 0.229 |
| **Patient mix_medical patients** | 65 (90.3%) | 35 (89.7 %) | 30 (90.9 %) | 0.868 |
| **Reasons for ICU admission** |  |  |  | 0.395 |
| Respiratory problems | 24 (33.3 %) | 15 (38.5 %) | 9 (27.3 %) |  |
| Cardiovascular problems | 8 (11.1 %) | 6 (15.4 %) | 2 (6.1 %) |  |
| Neurological problems | 4 (5.6 %) | 3 (7.7 %) | 1 (3 %) |  |
| Gastroenterological problems | 5 (6.9 %) | 1 (2.6 %) | 4 (12.1 %) |  |
| Nephrological problem | 12 (16.7 %) | 5 (12.8 %) | 7 (21.2 %) |  |
| **With infection ^＃^** | 39 (54.2 %) | 6 (15.4 %) | 33 (100 %) | <0.001 |
| **Types of infection *** |  |  |  |  |
| Pneumonia | 10 (13.9 %) | 0 (0.0 %) | 10 (30.3 %) | <0.001 |
| Urinary tract infection | 11 (15.3 %) | 1 (2.6 %) | 10 (30.3 %) | <0.001 |
| Blood stream infection | 23 (31.9 %) | 4 (10.3 %) | 19 (57.6 %) | <0.001 |
| Skin infection | 4 (5.6 %) | 1 (2.6 %) | 3 (9.1 %) | 0.228 |
| Other infection | 9 (12.5 %) | 2 (5.1 %) | 7 (21.2 %) | 0.040 |

**Note:** Continuous variables with normal distribution were reported as mean ± standard deviation and compared using an independent t-test. Those with abnormal distribution were presented with median (range) and compared using an independent t-test after log transformation and confirmation as normal distribution by Q-Q plot. Categorical variables were expressed as case number (percentage) and compared using the chi-square test.

^＃^ suspected or documented infection

***** we counted all types of infection if a patient had more than one type of infection.

**Abbreviations:** SOFA= sequential organ failure assessment.
