# Supplementary material for: A novel procalcitonin-based score for detecting sepsis among critically ill patients
Source: PLoS One. 2021 Jan 22;16(1):e0245748. doi: 10.1371/journal.pone.0245748 (PMC7822524; doi:10.1371/journal.pone.0245748)
Supplement: S4 Table — Abbreviations: IPS = infection probability score; PaO2/FiO2 = partial pressure of oxygen divided by the fraction of inspired oxygen; PCT = procalcitonin; SOFA = sequential organ failure assessment. (DOCX) [file pone.0245748.s005.docx]

**S4 Table. Comparisons of the components in the scores**

| Variables | **Current PCT-based score** | **SOFA score** | **IPS** |
| --- | --- | --- | --- |
| Procalcitonin | **V** | --- | --- |
| Albumin | **V** | --- | --- |
| Neutrophils-Lymphocyte ratio | **V** | --- | --- |
| Diabetes mellitus | **V** | --- | --- |
| Vasopressor | **V** | **V** | **V** |
| Mean arterial pressure |  | **V** | **V** |
| Creatinine |  | **V** | **V** |
| PaO2/FiO2 | --- | **V** | **V** |
| Total bilirubin | --- | **V** | **V** |
| Glasgow coma scale | --- | **V** | **V** |
| Platelet | --- | **V** | **V** |
| Heart rate | --- | --- | **V** |
| Respiratory rate | --- | --- | **V** |
| Body temperature | --- | --- | **V** |
| White blood cell | --- | --- | **V** |
| C-reactive protein | --- | --- | **V** |

**Abbreviations:** IPS= infection probability score; PaO2/FiO2= partial pressure of oxygen divided by the fraction of inspired oxygen; PCT= procalcitonin; SOFA= sequential organ failure assessment.
